# Neurological Pupil Index for the Early Prediction of Outcome in Severe Acute Brain Injury Patients

**DOI:** 10.3390/brainsci12050609

**Published:** 2022-05-06

**Authors:** Federico Romagnosi, Adriano Bernini, Filippo Bongiovanni, Carolina Iaquaniello, John-Paul Miroz, Giuseppe Citerio, Fabio Silvio Taccone, Mauro Oddo

**Affiliations:** 1 Neuroscience Critical Care Group, Department of Intensive Care Medicine, Centre Hospitalier Universitaire Vaudois (CHUV), Lausanne University Hospital, University of Lausanne, 1011 Lausanne, Switzerland; federico.romagnosi@gmail.com (F.R.); bernini.adriano@googlemail.com (A.B.); filippo.bongiovanni02@gmail.com (F.B.); ca.iaquaniello@gmail.com (C.I.); john-paul.miroz@chuv.ch (J.-P.M.); 2 Department of Anesthesiology and Intensive Care Medicine, University of Verona, 37124 Verona, Italy; 3 Department of Anesthesiology and Intensive Care Medicine, Catholic University of the Sacred Heart, 00168 Roma, Italy; 4 Department of Neuroanesthesia and Neurointensive Care, Istituto Neurologico “Carlo Besta”, 20133 Milano, Italy; 5 Department of Neuroscience ASST-Monza, University of Milano—Bicocca, 20900 Monza, Italy; giuseppe.citerio@unimib.it; 6 Department of Intensive Care Medicine, Erasme Hospital, Université Libre de Bruxelles, 1050 Brussels, Belgium; fabio.taccone@ulb.be; 7 Medical Directorate for Research, Education and Innovation, Direction Médicale, Centre Hospitalier Universitaire Vaudois (CHUV), University of Lausanne, 1011 Lausanne, Switzerland

**Keywords:** Neurological Pupil index, acute brain injury, quantitative pupillometry, neurological prognosis, midline shift

## Abstract

In this study, we examined the early value of automated quantitative pupillary examination, using the Neurological Pupil index (NPi), to predict the long-term outcome of acute brain injured (ABI) patients. We performed a single-centre retrospective study (October 2016–March 2019) in ABI patients who underwent NPi measurement during the first 3 days following brain insult. We examined the performance of NPi—alone or in combination with other baseline demographic (age) and radiologic (CT midline shift) predictors—to prognosticate unfavourable 6-month outcome (Glasgow Outcome Scale 1–3). A total of 145 severely brain-injured subjects (65 traumatic brain injury, TBI; 80 non-TBI) were studied. At each time point tested, NPi <3 was highly predictive of unfavourable outcome, with highest specificity (100% (90–100)) at day 3 (sensitivity 24% (15–35), negative predictive value 36% (34–39)). The addition of NPi, from day 1 following ABI to age and cerebral CT scan, provided the best prognostic performance (AUROC curve 0.85 vs. 0.78 without NPi, *p* = 0.008; DeLong test) for 6-month neurological outcome prediction. NPi, assessed at the early post-injury phase, has a superior ability to predict unfavourable long-term neurological outcomes in severely brain-injured patients. The added prognostic value of NPi was most significant when complemented with baseline demographic and radiologic information.

## 1. Introduction

Pupillary reactivity is an important component of the neurological assessment of patients with acute brain injury (ABI) [1,2,3,4], with robust prognostic performance [5,6]. An abnormal pupillary function is generally considered an early sign of neurological deterioration [7,8] and worsening intra-cerebral lesion with impending brain herniation [9]. Despite its known and validated prognostic implications, the clinical evaluation of pupillary reactivity mainly relies on subjective assessment using qualitative tools [10], which may lead to inaccuracies, especially in the early phase after the initial insult, when patients are under sedation and analgesia as part of ABI management [11]. Recently, automated infrared pupillometry has been implemented in critical care to obviate the limitations of standard qualitative assessment [12], either as a complementary monitoring tool of secondary cerebral damage (e.g., intracranial hypertension [13] and delayed ischemia [14]) or as part of multimodal prognostication of patients with hypoxic-ischemic brain injury following cardiac arrest [15,16,17], using the Neurological Pupil index (NPi) as the outcome predictor. Quantitative pupillometry has increasingly validated prognostic value in the setting of cardiac arrest care [18]; however, its value for the prognostication of coma following non-anoxic ABI is not fully established.

We, therefore, designed this study to investigate the value of automated quantitative NPi in predicting the outcome of high-risk (due to potential worsening of the intra-cerebral lesion) critically ill, severely brain-injured patients.

## 2. Materials and Methods

### 2.1. Study Design

This single-centre retrospective analysis was performed from October 2016 to March 2019 at the Department of Intensive Care Medicine, Centre Hospitalier Universitaire Vaudois (CHUV), Lausanne University Hospital, Switzerland. We included all consecutive adult (age ≥ 18 years) non-anoxic ABI patients who underwent repeated NPi measurements (using the NPi-200 pupillometer^®^, NeurOptics, Laguna Hills, CA, USA), as routine care.

Patients with ABI after cardiac arrest were excluded from the present study. Additional exclusion criteria included unavailable neurological assessment at six-month follow-up, unavailable data during the first 72 h post-injury, and previously known ophthalmic conditions (i.e., facial and ocular injuries, prosthetic eyes), which cause unreliable pupillary assessment. The project was approved by the Ethical Research Committee of the University of Lausanne, with a waiver of informed consent because of the retrospective design, and used de-identified electronic data extraction; all data were part of standard care.

The study report conforms to the Standards for Reporting Diagnostic accuracy studies (STARD) 2015 guidelines for prognostic accuracy studies [19].

### 2.2. Automated Quantitative Pupillometry

The NPi^®^-200 pupillometer (NeurOptics, Laguna Hills, CA, USA) allows quantitative measurement of different pupil variables (i.e., pupil size, percentage of constriction, the latency of constriction, constriction velocity, dilation velocity). Based on the integration of these variables, the device can compute the NPi, a scalar value (ranging from 0 to 5 ± 0.1), derived from a proprietary algorithm. Each NPi measurement was performed on both patients’ eyes and in stable ambient light conditions to avoid interferences [12]. The lowest value measured between the two eyes was retained for analysis. Assessment of the NPi was performed at least 3 times per day by the nurse in charge of the patient, as part of standard care. For each patient, the mean NPi and the percentage of abnormal NPi (defined as below 3, in line with reported standards [20] and previous publications [13]) were calculated daily, for the first 3 days. Data were extracted retrospectively from patients’ computerised medical records.

### 2.3. Patient Management

According to current guidelines, standardised, validated internal protocols [6,21,22,23] were applied to treat patients with severe ABI admitted to the intensive care unit (ICU). All patients were mechanically ventilated, aiming to keep PaO_2_ and PaCO_2_ between 90 and 100 mmHg and 36 and 40 mmHg, respectively. Early-phase analgosedation was guided by a written protocol, using propofol (maximal dose, 4 mg/kg/h) and sufentanil (maximal dose, 20 μg/h). Targeted systemic control included maintenance of cerebral perfusion pressure >60–70 mmHg (aiming at euvolemia and using norepinephrine when needed), arterial blood glucose at 6–8 mmol/L (with the use of continuous insulin infusion), normothermia (core body temperature  < 37.8 °C), and early institution of enteral nutrition. Management of elevated intracranial pressure (ICP) followed a stepwise management algorithm [13], consisting of reinforced sedation (increased propofol ± midazolam), moderate hyperventilation (PaCO_2_ 30–35  mmHg), and reinforced temperature management (35–37 °C, targeted to ICP control). Osmotherapy consisted of 7.5% hypertonic saline (2 mL/kg) or 20% mannitol (0.5 g/kg).

### 2.4. Radiological and Outcome Assessment

During the ICU stay, cerebral CT scans were performed by CHUV radiologists. Post-resuscitation cerebral CT scan on day 1, from ICU admission, was assessed for midline shift (expressed in mm, calculated by measuring the perpendicular distance between the septum pellucidum and the midline, drawing a line between the anterior and posterior attachment of the falx) from radiologist reports. Abnormal midline shift was defined as ≥5 mm [24].

The 6-month neurological outcome was assessed as part of routine care through a face-to-face interview by a neurosurgeon, using the Glasgow Outcome Scale (GOS), dichotomised as unfavourable (GOS 1–3) or favourable neurological recovery (GOS 4–5).

Cerebral CT scan and outcome assessments were performed blinded to NPi data.

### 2.5. Data Collection and Analysis

Patient baseline demographic variables included age, gender, ABI diagnosis (dichotomised as traumatic brain injury (TBI) vs. non-TBI, i.e., including aneurysmal subarachnoid haemorrhage, intracranial haemorrhage, and ischaemic stroke), on-site Glasgow coma scale (GCS), and ICU length of stay (LOS).

Descriptive data are presented as mean and 95% confidence interval (95% CI), for continuous variables, and as counts (percentage) for categorical variables. Data distribution was assessed with the Shapiro–Wilk test. Comparisons between variables were performed with non-parametric Wilcoxon–Mann–Whitney and Fisher tests. The Association of NPi with the outcome variable (6-month GOS) was analysed with the non-parametric Wilcoxon test. Prognostic performance was analysed for the Neurological Pupil index (NPi) test and the reference standard (patient age, CT midline shift), by calculating the specificity, sensitivity, positive predictive value (PPV), negative predictive value (NPV), and the area under the receiver operating characteristic (AUROC) curve. The AUROC curve comparisons for single tests and combined tests (age and CT midline shift with or without NPi) were analysed using the DeLong test [25]. Statistical analysis was performed with JMP 15.1.0 (SAS Institute Inc., Cary, NC, USA). All analyses were set with significance at *p* < 0.05.

## 3. Results

### 3.1. Patient Characteristics

**Figure 1:**
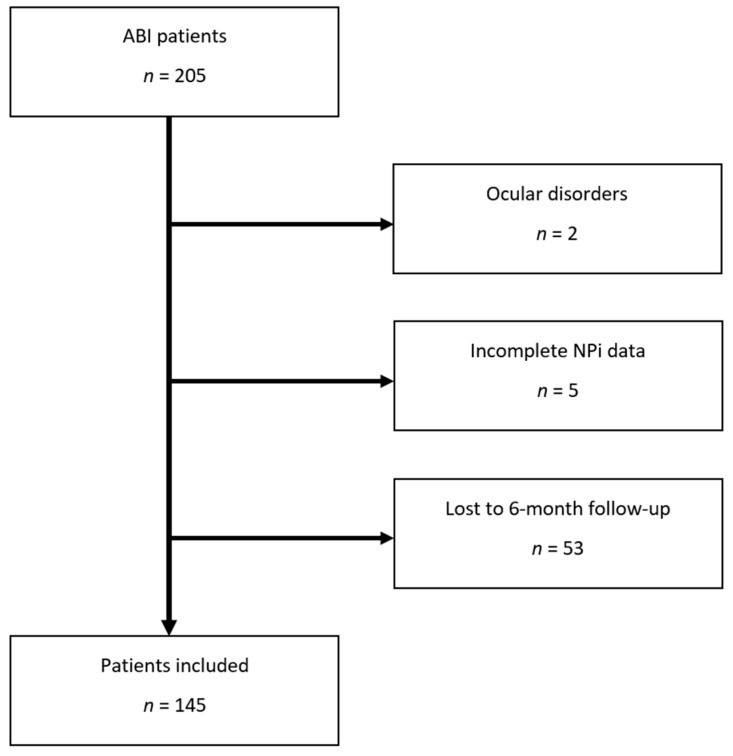
Study flowchart. Abbreviations: NPi: Neurological Pupil index.

Out of 205 consecutive ABI patients, 60 were excluded (53 without 6-month outcome follow-up, 5 with incomplete NPi data, and 2 with eye disorders making pupillometry assessment unreliable), leaving a total of 145 patients for further data analysis (study flowchart, Figure 1). Among these, 65 (45%) had TBI, and 80 (55%) had non-TBI diagnoses, including ischemic–haemorrhagic stroke (*n* = 38, 26%), subarachnoid haemorrhage (*n* = 30, 21%) and other ABI diagnoses (infectious encephalitis and cerebral venous thrombosis: *n* = 12, 8%). Unfavourable neurological outcome was observed in 103 (71%) patients (Table 1).

### 3.2. Outcome Associations

**Figure 2:**
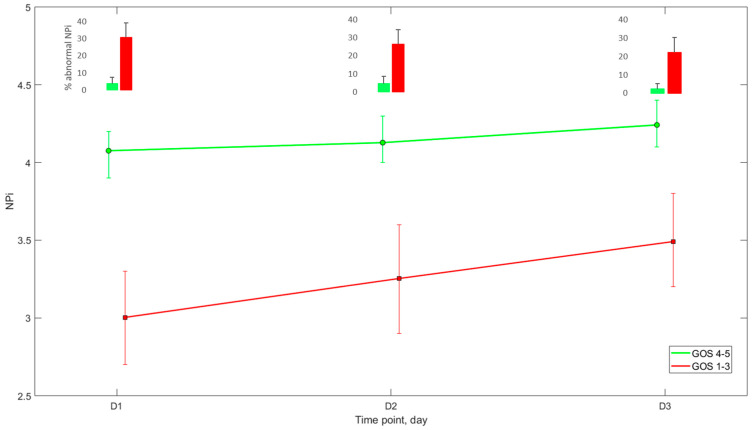
NPi pattern during the first 3 days after ICU admission according to 6-month Glasgow Outcome Score (mean + 95%CI). Abbreviations: NPi = Neurological Pupil index, GOS: Glasgow outcome scale, ICU: intensive care unit.

As shown in Table 1 and Figure 2, NPi on day 1 to 3 was significantly lower in patients with unfavourable outcome than those with favourable neurological outcome, (day 1: 3 (2.7–3.3) vs. 4.1 (3.9–4.2), *p* = 0.009; day 2: 3.3 (2.9–3.6) vs. 4.1 (4–4.3), *p* = 0.051; day 3: 3.5 (3.2–3.8) vs. 4.2 (4.1–4.4), *p* = 0.06), while the percentage of abnormal NPi recordings was significantly higher (day 1: 31 (22.5–39)% vs. 4 (0–8)%, *p* = 0.001; day 2: 26.5 (18.5–34.5)% vs. 4.5 (0.5–9)%, *p* = 0.003; day 3: 22 (14–30)% vs. 2 (0–5)%, *p* = 0.004). Midline shift was more frequent (47%) and greater in patients with unfavourable neurological outcome, when compared with the others (4.9 (3.9–5.9) vs. 2.0 (1.1–3) mm. *p* = 0.003; Table 1). Notably, age was related to unfavourable long-term neurological outcome (60 ± 16 vs. 46 ± 18 years old; *p* < 0.001).

### 3.3. Prognostic Performance of NPi

As reported in Table 2, NPi below 3 at each time point had a good accuracy to predict neurological outcomes, reaching the highest specificity (100 (90–100)%) and PPV (100%) on day 3, while sensitivity (34 (25–44)%) and NPV (38 (34–41)%) were overall low. Compared with NPi, abnormal midline shift on CT scan had lower specificity (83% (69–93)%) and higher sensitivity (47% (37–57)%) for unfavourable neurological outcome prediction.

**Figure 3:**
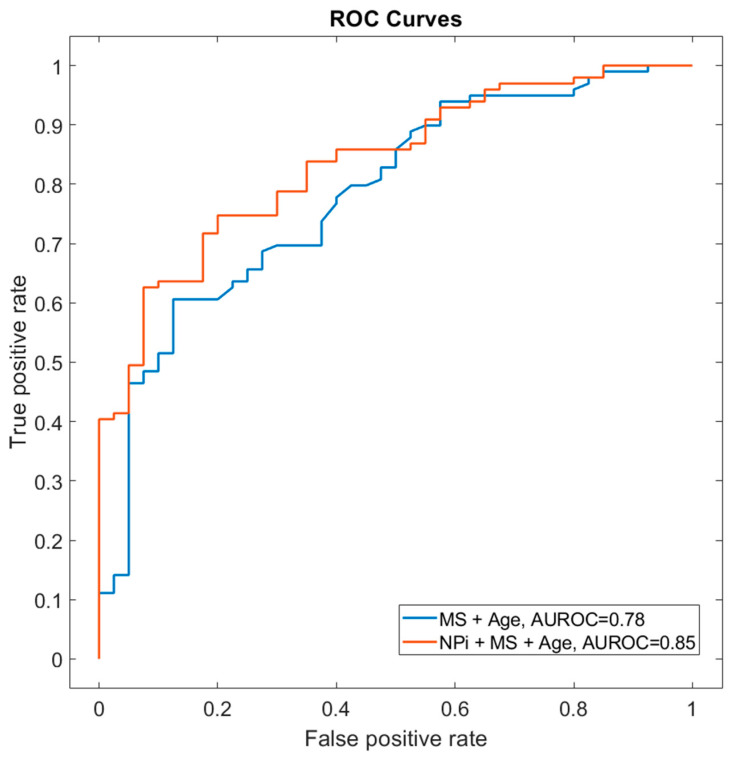
Prognostic performance of NPi and midline shift at day 1. The receiver operator characteristic curve (AUROC) shows the prognostic performance scores using the combination of MS + age (AUROC curve, 0.78) in comparison with NPi day 1 + MS + age (AUROC curve, 0.85; DeLong test *p* = 0.008). Abbreviations: AUROC: area under receiver operating characteristics, MS: midline shift, NPi: Neurological Pupil index.

The prognostic performance of NPi was comparable to that of CT midline shift (AUROC = 0.64 vs. 0.66) and lower than that of age (AUROC = 0.75). A model combining NPi, midline shift (both on day 1) and age provided the best prognostic performance (AUROC = 0.85 vs. 0.78 without NPi, *p* = 0.008 DeLong test, Figure 3).

## 4. Discussion

The main findings of this single-centre retrospective study conducted in non-anoxic ABI patients can be summarised as follows: First, low NPi values—when taken alone and using a cut-off of less than 3 for outcome prognosis—is a strong predictor of unfavourable neurological outcome, with very high specificity and positive predictive value during the early-phase post-injury. Second, when combined with available baseline demographic (age) and radiologic (cerebral CT midline shift) prognosticators, clinical pupillary assessment with the use of automated NPi already on day 1 significantly improved the prediction of long-term neurological outcomes after ABI.

In our study, patients with unfavourable 6-month neurological outcomes had lower NPi over the first three days from ICU admission (NPi 3.1 vs. 4.1). When we focused on NPi values every single day, we observed that, on day 1, the NPi difference between favourable and unfavourable outcome populations was the highest, decreasing over the following two days (Table 2, Figure 2). These findings suggest a prognostic value of the NPi in the first 24 h from admission in predicting unfavourable neurological outcomes at 6 months. It is noteworthy that patients with adverse outcomes had significantly higher percentages of abnormal NPi (below 3) when compared with their favourable outcome counterparts. From a practical standpoint, these data suggest that NPi should be regarded as a non-invasive monitoring tool for patients with severe brain lesions, mostly basing clinical outcome prediction on the overall burden of pathological NPi rather than on single observations: in this setting, the persistence of pathological pupillary indices should be regarded as a sign of ongoing brain damage, thus outlining an unfavourable outcome trajectory.

On the one hand, when analysing the prognostic role of NPi, using a cut-off of 3 to define abnormal values, we found it to have a superior ability to predict an unfavourable 6-month outcome at each time point tested from day 1 to 3, with the highest specificity (100% [90–100%]) and PPV (100%) on day 3. On the other hand, NPi had low sensitivity, aligned with previous observations in hypoxic brain injury in comatose cardiac arrest patients [15].

NPi is best used in combination with other prognostic variables, applying a multimodal assessment with multiple predictors [18]. In our study, we combined clinical data (pupillary reactivity using automated quantitative NPi) with baseline demographic (age) and radiologic (cerebral CT midline shift) variables, both of which are well-known robust outcome predictors [26]. A model combining patient age and cerebral CT midline shift returned a prognostic value AUROC of 0.78. Moreover, the addition of NPi indeed significantly increased the ability to predict unfavourable neurological outcomes in our single-centre cohort (AUROC 0.85; Figure 3).

### Study Limitations

Our study has several limitations. First, findings are limited by the single-centre retrospective design of the study and the high grade of injury severity of the cohort, composed mainly of patients in whom cerebral CT scan showed intracranial lesions and evidence of cerebral oedema, thereby explaining the high rate of mortality and unfavourable neurological outcomes. The generalisability of our findings is, therefore, limited and additional multicentre confirmatory studies are needed [27]. Second, the cohort comprised a heterogeneous group of patients with different primary brain insults, each having different pathophysiologic mechanisms and injury pathways that could hamper the interpretation of the results. Due to this aspect, although the presence of CT midline shift is a well-established outcome predictor [1], we could not test prognostic radiologic scores, such as the Marshall [28] or the Fisher scores [29]. Third, we did not perform a more detailed logistic regression analysis to potentially identify specific patterns’ NPi and their association with patient outcomes. Fourth, we restricted our analysis to the first three days following ABI, without additional insights into NPi trajectories over time across outcome groups. This choice is in line with our previous study in cardiac arrest patients [15] and, from a methodological standpoint, allowed the analysis of a homogeneous NPi dataset at the early phase of ABI, obtained standardised conditions of sedation and analgesia, aiming at minimising a potential impact of sedative and analgesic dose on NPi [30]. Fifth, outcome associations and prognostic performance of quantitative pupillometry data were restricted to NPi—a calculated parameter derived from the integration of multiple measured pupillary variables—but we did not assess the value of single pupillary variables, such as the percentage pupillary constriction or constriction velocity. Finally, and most importantly, self-fulfilling prophecy is a significant limitation, as with all prognostic studies. To overcome such a limitation, at least partly, NPi data were not part of clinical decision making and were blinded to radiologic and outcome assessors.

## 5. Conclusions

Abnormal NPi, assessed at the early post-injury phase, has very high specificity to predict unfavourable six-month outcomes in ABI patients with high-grade CT scan lesions. The added prognostic value of NPi was most significant when complemented with baseline demographic (age) and radiologic (CT midline shift) information. Our findings highlight the importance of integrated multimodal assessment of ABI prognosis and support the value of quantitative pupillometry as an additional prognosticating tool in this setting.

## Figures and Tables

**Table 1:** Patient characteristics.

Variable	Favourable Outcome (GOS 4–5) N = 42	Unfavourable Outcome (GOS 1–3) N = 103 *	*p* Value
Age, years	46 ± 18	60 ± 16	<0.0001
Female gender, *n* (%)	16 (38)	47 (46)	0.46
ICU length of stay, days	10 [8–12]	11 [10–12]	0.516
GCS on site	7 ± 3	7 ± 4	0.93
TBI vs. non-TBI, *n* (%)	26 (62)	39 (38)	0.01
Midline shift on head CT ≥ 5 mm, *n* (%)	7 (17)	48 (47)	0.0008
Midline shift on head CT, mm	2.0 [1.1–3]	4.9 [3.9–5.9]	0.003
NPi *, day 1	4.1 [3.9–4.2]	3.0 [2.7–3.3]	0.009
NPi *, day 2	4.1 [4–4.3]	3.3 [2.9–3.6]	0.051
NPi *, day 3	4.2 [4.1–4.4]	3.5 [3.2–3.8]	0.06
Abnormal NPi < 3 (%), day 1	4 [0–8]	31 [22.5–39]	0.0001
Abnormal NPi < 3 (%), day 2	4.5 [0.5–9]	26.5 [18.5–34.5]	0.003
Abnormal NPi < 3 (%), day 3	2 [0–5]	22 [14–30]	0.0039

Data are presented as percentages, mean with standard deviations, or 95% confidence intervals. * Mean daily NPi value. Abbreviations: GCS: Glasgow Coma Scale, GOS: Glasgow Outcome Scale, NPi: Neurological Pupil index, TBI: traumatic brain injury.

**Table 2:** Prognostic performance of single tests.

Variable	Patient Number	Unfavourable Outcome (GOS 1–3) at 6 Months, *n* (%)	Specificity % (95% CI)	Sensitivity % (95% CI)	PPV (95% CI)	NPV (95% CI)
Neurological Pupil index (NPi) < 3						
Day 1–3 post-admission	145	103 (71)	98 (87–100)	34 (25–44)	97 (83–100)	38 (34–41)
Day 1	139	99 (71)	97 (85–100)	33 (24–44)	97 (82–100)	33 (30–37)
Day 2	132	94 (71)	97 (86–100)	31 (22–41)	97 (80–100)	36 (33–40)
Day 3	119	83 (70)	100 (90–100)	24 (15–35)	100	36 (34–39)
Head CT scan showing ≥ 5 mm midline shift						
Day1	145	103 (71)	83 (69–93)	47 (37–57)	87 (77–93)	39 (34–44)

Abbreviations: CI: confidence interval, GOS: Glasgow Outcome Scale, NPi: Neurological Pupil index, NPV: negative predictive value, PPV: positive predictive value.

## Data Availability

The datasets used and analysed during the current study are available from the corresponding author on reasonable request.

## References

[1] Marmarou A., Lu J., Butcher I., McHugh G.S., Murray G.D., Steyerberg E.W., Mushkudiani N.A., Choi S., Maas A.I.R. (2007). Prognostic Value of The Glasgow Coma Scale And Pupil Reactivity in Traumatic Brain Injury Assessed Pre-Hospital And on Enrollment: An IMPACT Analysis. J. Neurotrauma.

[2] Majdan M., Steyerberg E.W., Nieboer D., Mauritz W., Rusnak M., Lingsma H.F. (2015). Glasgow Coma Scale Motor Score and Pupillary Reaction To Predict Six-Month Mortality in Patients with Traumatic Brain Injury: Comparison of Field and Admission Assessment. J. Neurotrauma.

[3] Stevens R.D., Sutter R. (2013). Prognosis in Severe Brain Injury. Crit. Care Med..

[4] Han J., King N.K.K., Neilson S.J., Gandhi M.P., Ng I. (2014). External Validation of the CRASH and IMPACT Prognostic Models in Severe Traumatic Brain Injury. J. Neurotrauma.

[5] Nolan J.P., Cariou A. (2015). Post-Resuscitation Care: ERC–ESICM Guidelines 2015. Intensive Care Med..

[6] Carney N., Totten A.M., O’Reilly C., Ullman J.S., Hawryluk G.W.J., Bell M.J., Bratton S.L., Chesnut R., Harris O.A., Kissoon N. (2017). Guidelines for the Management of Severe Traumatic Brain Injury, Fourth Edition. Neurosurgery.

[7] Chesnut R.M., Gautille T., Blunt B.A., Klauber M.R., Marshall L.E. (1994). The Localizing Value of Asymmetry in Pupillary Size in Severe Head Injury: Relation to Lesion Type and Location. Neurosurgery.

[8] Chesnut R., Aguilera S., Buki A., Bulger E., Citerio G., Cooper D.J., Arrastia R.D., Diringer M., Figaji A., Gao G. (2020). A Management Algorithm for Adult Patients with Both Brain Oxygen and Intracranial Pressure Monitoring: The Seattle International Severe Traumatic Brain Injury Consensus Conference (SIBICC). Intensive Care Med..

[9] Ritter A.M., Muizelaar J.P., Barnes T., Choi S., Fatouros P., Ward J., Bullock M.R. (1999). Brain Stem Blood Flow, Pupillary Response, and Outcome in Patients with Severe Head Injuries. Neurosurgery.

[10] Couret D., Boumaza D., Grisotto C., Triglia T., Pellegrini L., Ocquidant P., Bruder N.J., Velly L.J. (2016). Reliability of Standard Pupillometry Practice in Neurocritical Care: An Observational, Double-Blinded Study. Crit. Care.

[11] Olson D.M., Stutzman S., Saju C., Wilson M., Zhao W., Aiyagari V. (2016). Interrater Reliability of Pupillary Assessments. Neurocritical Care.

[12] Morelli P., Oddo M., Ben-Hamouda N. (2019). Role of Automated Pupillometry in Critically Ill Patients. Minerva Anestesiol..

[13] Jahns F.-P., Miroz J.P., Messerer M., Daniel R.T., Taccone F.S., Eckert P., Oddo M. (2019). Quantitative Pupillometry for the Monitoring of Intracranial Hypertension in Patients with Severe Traumatic Brain Injury. Crit. Care.

[14] Aoun S.G., Stutzman S.E., Vo P.-U.N., Ahmadieh T.Y.E., Osman M., Neeley O., Plitt A., Caruso J.P., Aiyagari V., Atem F. (2019). Detection of Delayed Cerebral Ischemia Using Objective Pupillometry in Patients with Aneurysmal Subarachnoid Hemorrhage. J. Neurosurg..

[15] Oddo M., Sandroni C., Citerio G., Miroz J.-P., Horn J., Rundgren M., Cariou A., Payen J.-F., Storm C., Stammet P. (2018). Quantitative versus Standard Pupillary Light Reflex for Early Prognostication in Comatose Cardiac Arrest Patients: An International Prospective Multicenter Double-Blinded Study. Intensive Care Med..

[16] Riker R.R., Sawyer M.E., Fischman V.G., May T., Lord C., Eldridge A., Seder D.B. (2019). Neurological Pupil Index and Pupillary Light Reflex by Pupillometry Predict Outcome Early After Cardiac Arrest. Neurocritical Care.

[17] Tamura T., Namiki J., Sugawara Y., Sekine K., Yo K., Kanaya T., Yokobori S., Roberts R., Abe T., Yokota H. (2018). Quantitative Assessment of Pupillary Light Reflex for Early Prediction of Outcomes after Out-of-Hospital Cardiac Arrest: A Multicentre Prospective Observational Study. Resuscitation.

[18] Nolan J.P., Sandroni C., Böttiger B.W., Cariou A., Cronberg T., Friberg H., Genbrugge C., Haywood K., Lilja G., Moulaert V.R.M. (2021). European Resuscitation Council and European Society of Intensive Care Medicine Guidelines 2021: Post-Resuscitation Care. Resuscitation.

[19] Korevaar D.A., Cohen J.F., Reitsma J.B., Bruns D.E., Gatsonis C.A., Glasziou P.P., Irwig L., Moher D., de Vet H.C.W., Altman D.G. (2016). Updating Standards for Reporting Diagnostic Accuracy: The Development of STARD 2015. Res. Integr. Peer Rev..

[20] Chen J., Gombart Z., Rogers S., Gardiner S., Cecil S., Bullock R. (2011). Pupillary Reactivity as an Early Indicator of Increased Intracranial Pressure: The Introduction of the Neurological Pupil Index. Surg. Neurol. Int..

[21] Hemphill J.C., Greenberg S.M., Anderson C.S., Becker K., Bendok B.R., Cushman M., Fung G.L., Goldstein J.N., Macdonald R.L., Mitchell P.H. (2015). Guidelines for the Management of Spontaneous Intracerebral Hemorrhage: A Guideline for Healthcare Professionals From the American Heart Association/American Stroke Association. Stroke.

[22] Bederson J.B., Connolly E.S., Batjer H.H., Dacey R.G., Dion J.E., Diringer M.N., Duldner J.E., Harbaugh R.E., Patel A.B., Rosenwasser R.H. (2009). Guidelines for the Management of Aneurysmal Subarachnoid Hemorrhage: A statement for healthcare professionals from a special writing group of the Stroke Council. Stroke.

[23] Connolly E.S., Rabinstein A.A., Carhuapoma J.R., Derdeyn C.P., Dion J., Higashida R.T., Hoh B.L., Kirkness C.J., Naidech A.M., Ogilvy C.S. (2012). Guidelines for the Management of Aneurysmal Subarachnoid Hemorrhage: A guideline for healthcare professionals from the American Heart Association/American Stroke Association. Stroke.

[24] Puffer R.C., Yue J.K., Mesley M., Billigen J.B., Sharpless J., Fetzick A.L., Puccio A., Diaz-Arrastia R., Okonkwo D.O. (2018). Long-Term Outcome in Traumatic Brain Injury Patients with Midline Shift: A Secondary Analysis of the Phase 3 COBRIT Clinical Trial. J. Neurosurg..

[25] DeLong E.R., DeLong D.M., Clarke-Pearson D.L. (1988). Comparing the Areas under Two or More Correlated Receiver Operating Characteristic Curves: A Nonparametric Approach. Biometrics.

[26] Gao G., Wu X., Feng J., Hui J., Mao Q., Lecky F., Lingsma H., Maas A.I.R., Jiang J., China CENTER-TBI Registry Participants (2020). Clinical Characteristics and Outcomes in Patients with Traumatic Brain Injury in China: A Prospective, Multicentre, Longitudinal, Observational Study. Lancet Neurol..

[27] Oddo M., Taccone F., Galimberti S., Rebora P., Citerio G. (2021). Outcome Prognostication of Acute Brain Injury Using the Neurological Pupil Index (ORANGE) Study: Protocol for a Prospective, Observational, Multicentre, International Cohort Study. BMJ Open.

[28] Mohammadifard M., Ghaemi K., Hanif H., Sharifzadeh G., Haghparast M. (2018). Marshall and Rotterdam Computed Tomography Scores in Predicting Early Deaths after Brain Trauma. Eur. J. Transl. Myol..

[29] Lindvall P., Runnerstam M., Birgander R., Koskinen L.-O.D. (2009). The Fisher Grading Correlated to Outcome in Patients with Subarachnoid Haemorrhage. Br. J. Neurosurg..

[30] Larson M.D., Behrends M. (2015). Portable Infrared Pupillometry: A Review. Anesth. Analg..

